# Supplementary material for: A Systematic Umbrella Review of the Effects of Teledentistry on Costs and Oral-Health Outcomes
Source: Int J Environ Res Public Health. 2024 Mar 28;21(4):407. doi: 10.3390/ijerph21040407 (PMC11050059; doi:10.3390/ijerph21040407)
Supplement: Supplementary file 1 [file ijerph-21-00407-s001.zip › AMSTAR 2-Guidance-document.pdf]

AMSTAR 2 guidance document

Many of the items in AMSTAR 2 are written to be self-explanatory. However, the underlying issues are often complex, and subject to varying interpretation, particularly when judgments are made across a wide spectrum of interventions. Here we provide additional guidance on use of AMSTAR 2. Material in this document overlaps with that in the published paper. This is intentional, as this Appendix is intended to be a stand-alone document.

We emphasise this is guidance – it gives an indication of how we think the criteria should be applied in settings where reviews are conducted of well-defined (usually clinical) interventions. Individual users, of course, may find it necessary to deviate from the guidance both in addressing individual domains and in making an overall appraisal of a systematic review. We ask that in doing so they document these variations so that others can benefit from their experiences

AMSTAR 2 is not designed to generate an overall 'score'. A high score may disguise critical weaknesses in specific domains, such as an inadequate literature search or are a failure to assess risk of bias (ROB) with individual studies that were included in a systematic review. In making an overall rating of systematic review it is important to take account of flaws in critical domains, which may greatly weaken the confidence that can be placed in a systematic review.

Item 1: Did the research questions and inclusion criteria for the review include the components of PICO?

It is common practice to use PICO description (population, intervention, control group and outcome) as an organising framework for a study question. Sometimes timeframe should be added if this is critical in determining the likelihood of a study capturing relevant clinical outcomes (e.g. an effect of the intervention is only expected after several years). PICO identifies the elements that should be described in detail in the report of the systematic review and should enable the appraiser to judge selection of studies, and their combinability, and enable the user of the review to determine applicability of the results. Authors of systematic reviews do not always make the elements of PICO explicit but they should be discernable through a careful reading of the abstract, introduction and methods sections. To score 'Yes' appraisers should be confident that the 4 elements of PICO are described somewhere in the report.

Item 2: Did the report of the review contain an explicit statement that the review methods were established prior to conduct of the review and did the report justify any significant deviations from the protocol?

Systematic reviews are a form of observational research and the methods for the review should be agreed on before the review commences. Adherence to a well-developed protocol reduces the risk of bias in the review. Authors should demonstrate that they worked with a written protocol with independent verification. This can take the form of registration (e.g. at PROSPERO - https://www.crd.york.ac.uk/PROSPERO/), an open publication journal (e.g. BMJ Open) or a dated submission to a research office or research ethics board. The research questions and the review study methods should have been planned ahead of conducting the review. At a minimum this should be stated in the report (scores 'Partial Yes'). To score 'Yes' authors should demonstrate that they worked with a written protocol with independent verification (by a registry

or another independent body, e.g. research ethics board or research office) before the review was undertaken. Appraisers should compare the published report of the review with the registered protocol, when the latter is available. If there are deviations from the protocol appraisers should determine whether these are reported and justified by review authors. Obvious unexplained discrepancies should result in downgrading of the rating.

Item 3: Did the review authors explain their selection of the study designs for inclusion in the review?

The selection of study types for inclusion in systematic reviews should not be arbitrary. The authors should indicate that they followed a strategy. The general rule (this may have to be inferred from what the authors actually wrote) is that they asked first whether a review restricted to RCTs would have given an incomplete summary of the effects of a treatment. This might be because there were no relevant RCTs or because of missing outcomes in available RCTs [usually harms], inadequate statistical power, restrictive populations, or unrepresentative control/intervention treatments. If the answer to this general question is yes the inclusion of non-randomized studies of the intervention(s) is justified. Conversely, to justify restriction of the review to RCTs the authors should argue that they can provide a complete picture of the effects they are interested in. Restriction of a review to only NRSI is justified when RCTs cannot provide the necessary outcome data, or in the case where reviews of RCTs have been completed and the review of NRSI will complement what is already known. Inclusion of both RCTs and NRSI may be justified to get a complete picture of the effectiveness and harms associated with an intervention. In this situation we recommend (see below) that these two types of studies are assessed and combined independently (if meta-analysis is appropriate). This is a somewhat neglected area and even with guidance it can be difficult to judge the extent to which a review meets the rating criteria. The justification for selection of study designs may have to be inferred from a careful reading of the complete study report.

Item 4: Did the review authors use a comprehensive literature search strategy?

At least two bibliographic databases should be searched. The report should include years and databases examined (e.g., Central, EMBASE, and MEDLINE). Key words and/or MESH terms should be reported and the full search strategy available on request. Searches should be supplemented by checking published reviews, specialized registers, or experts in the particular field of study, and by reviewing the reference list from the studies found. Sometimes it is necessary to approach authors of original studies to clarify results or obtain updates or corrections. Publications in all relevant languages should be sought and a justification provided when there are language restrictions. We have highlighted the need for searching the grey literature in some cases. Grey literature is sometimes important with reports of policy and program evaluations that are only available from web sites (e.g. government, non-government or health technology agencies). These may or may not have been subject to peer review and such appraisals should be looked for. Where the grey literature is considered important, authors should have searched appropriate sources, such as trial registries, conference abstracts, dissertations, and unpublished reports on personal websites (e.g. universities, ResearchGate). In addition, trials of medical interventions may not have been published in peer-reviewed journals but can be obtained directly from company sponsors or directly from investigators. To score

‘Yes’ appraisers should be satisfied that all relevant aspects of the search have been addressed by review authors.

Item 5: Did the review authors perform study selection in duplicate?

Best practice requires two review authors to determine eligibility of studies for inclusion in systematic reviews. This involves checking the characteristics of a study (from title, abstract and full text) against the elements of the research question In the response options, we point to the desirability of review authors describing inter-rater agreement across a sample of studies being considered for inclusion in the review. A consensus process should have been used when disagreements arose in study selection. If one individual carried out selection of all studies, with a second reviewer checking agreement on a sample of studies, we recommend that a Kappa score indicating ‘strong’ agreement (0.80 or greater) should have been achieved. There should have been at least two independent assessors for study selection. A consensus process should have been used when disagreements arose in study selection. In the event that one individual carried out selection of studies a second reviewer should have checked agreement on a sample of representative studies and they should have achieved a kappa score of 0.80 or greater.

Item 6: Did the review authors perform data extraction in duplicate?

As in Item 5, there should have been at least two independent assessors performing data extraction. A consensus process should have been used when disagreements arose. In the event that one individual carried out data extraction a second reviewer should have checked agreement on a sample of studies and they should have achieved a kappa score of 0.80 or greater.

Item 7: Did the review authors provide a list of excluded studies and justify the exclusions?

This item requires review authors to provide a complete list of potentially relevant studies with justification for the exclusion of each. Non-inclusion of studies may be necessary for a range of reasons, based on inappropriate/ irrelevant populations, interventions and controls. Exclusion should not be based on risk of bias, which is dealt with separately and later in the review process. Unjustified exclusion may bias the review findings and we encourage an inclusive approach in the early stages of a review. This item requires review authors to provide a complete list of potentially relevant studies with justification for the exclusion of each one.

Item 8: Did the review authors describe the included studies in adequate detail?

The description of subjects, interventions, controls, outcomes, design, analysis and settings of the studies should be provided. The detail should be sufficient for an appraiser, or user, to make judgments about the extent to which the studies were appropriately chosen (in relation to the PICO structure) and whether the study populations and interventions were relevant their own practice or policy. The descriptors also provide a framework for studying heterogeneity in intervention effects (e.g. by dose, age range, clinical setting etc.)

Item 9: Did the review authors use a satisfactory technique for assessing the risk of bias (RoB) in individual studies that were included in the review?

This is a crucial part of the appraisal of any systematic review, particularly those that include non-randomized studies of interventions (NRSI). The key appraisal question is whether review authors have taken account of the risk of bias when summarising and interpreting the results.

When the review is confined to randomized controlled trials (RCTs) we recommend that you consult the Cochrane Handbook to determine whether the review authors have made an adequate assessment of ROB with individual RCTs. This section is concerned with the challenge posed by RoB in non-randomised studies.

Review authors should have used a systematic approach to ROB assessment, preferably with a properly developed rating instrument. If they have used a non-standard instrument you should be satisfied that it was capable of detecting serious methodological flaws. Several ROB instruments (for individual studies) are in common use, including the Newcastle Ottawa Scale, SIGN, and the Mixed Methods Appraisal Tool (MMAT). The most comprehensive assessment instrument is the recently introduced Cochrane instrument, ROBINS-I. It is appreciated that this instrument may not have been available at the time a review was performed.

In developing AMSTAR 2 we drew on the Cochrane RoB instruments for RCTs: (http://handbook.cochrane.org/chapter_8/8_5_the_cochrane_collaborations_tool_for_assessing_risk_of_bias.htm) and NRSI: www.riskofbias.info. In both cases the domain appraisal items are drawn from these instruments. Whatever instrument was used by the review authors, appraisers should be satisfied that it addresses the items listed in item 9 of the instrument.

Please note that the guidance given here is not comprehensive – AMSTAR 2 addresses only the most commonly recognised domains of bias. A deeper assessment of risk of bias requires specialist input. In assessing how RoB has been assessed by review authors you should seek methods and content expert advice, if that is not included in your team. Advisors should be asked to provide specific advice on which confounders are important, how to identify selection and measurement biases that are likely, to be relevant to the review under consideration. In addition, you should seek guidance on what adjustment techniques for confounding would be appropriate.

The following list of domains of bias has been selected from the ROBINS-I/ACROBAT-NRSI instrument as being the most relevant to systematic reviews that include NRSI:

Confounding. Confounding occurs when the effects of two associated interventions or exposures (e.g. smoking and alcohol consumption) have not been separated during analysis. This can result in an effect being attributed to one variable when it is due to the other. In the study of interventions confounding may also be related to the indication for treatment, for instance when one drug is given preferentially to patients with higher rates of co-morbidities than the comparator drug and where these co-morbid conditions are associated with the outcome of interest. These assessments are typically quantified in the baseline data reported in the individual study. Potential baseline confounding can be addressed in several ways, including design (eg matching by propensity score), adjustment (e.g. logistic regression) and other techniques such as instrumental variable analysis and the inclusion of 'tracer' exposures. It is common to assume that even sophisticated techniques will not adjust completely for all confounders, meaning weak associations, even if statistically significant, should be interpreted cautiously.

Sample selection bias. This occurs when subjects are sampled in a biased way that *directly* distorts the true relationship between exposure and outcome. It requires no third

factor, as is the case with confounding. For instance, you should not study the association between smoking and heart disease by recruiting subjects referred to a smoking cessation clinic. The selection of subjects with the exposure of interest should be unrelated to their outcome. Likewise, selection of subjects with the outcome should be unrelated to their exposure status. The timing of selection can be important. If subjects have been using a drug for some time before enrolment (prevalent users) they will be a tolerant group with a lower risk of adverse outcomes. For this reason, contemporary pharmaco-epidemiological studies recruit 'new users' of medications (analogous to starting treatment in a RCT). Other temporal sampling biases (immortal time bias and inception bias) are sometimes important. It is recommended that users refer to ROBINS-I guidance document for more information.

Bias in measurement of exposures and outcomes: measurement of an exposure or treatment may be misclassified if there is no accurate recording made in real time. Typically, modern pharmaco-epidemiological studies use prescriptions or dispensing records as a surrogate for consumption. But adherence to dispensed treatment will not be 100% so actual consumption will be miss-classified by this method. If this error is non-differential it will be a bias to the null. However, in some fields of research investigators rely on recall (e.g. ultraviolet exposure and melanoma). This may lead to differential misclassification. For instance, parents of a child who has died of SIDS may have heightened recall of any medications they administered to the baby prior to the event. Measurement of outcomes can also be affected by misclassification and if this is non-differential it will usually be a bias to the null. However, non-differential misclassification can introduce bias. For instance, if leg ultrasound is performed frequently in women with swollen painful legs who are taking an oral contraceptive, selection of individuals from an ultrasound clinic may bias studies of the association between DVT and oral contraceptives.

Selective reporting of outcomes and analyses: large observational studies may analyse population databases that record many outcomes occurring in a defined population. If outcomes are not pre-specified (preferably in a registered protocol) investigators may be tempted to analyse multiple outcomes and selectively report those that appear to be different between exposed and non-exposed individuals. In addition, there are usually several potential methods for analysing a non-randomized dataset (including, for example, different ways of categorising the intervention, or different multi-variable adjustment models). If the analytical protocol is not specified in advance of the study it may be possible to select one set of analyses that appears to show a significant statistical difference that is not apparent in the other analyses. Reviewers should determine whether study authors pre-specified outcomes and analyses. This will become easier as more studies are registered before being conducted.

Item 10: Did the review authors report on the sources of funding for the studies included in the review?

Several investigations have shown that commercially sponsored studies are more likely to have findings that favour a sponsor's product than independently funded studies. It is valuable for

review authors to document the funding sources for each study included in the review or to record that the information was not provided in the study reports. Depending on this information it may be possible to analyse separately the results from commercially funded and independently funded studies.

Item 11: If meta-analysis was justified did the review authors use appropriate methods for statistical combination of results? (Only complete this item if meta-analysis of other data synthesis techniques were reported)

Review authors should have stated explicitly in the review protocol the principles on which they based their decision to perform meta-analysis of data from the included studies. These include the desire to obtain a single pooled effect (for instance from a number of compatible but underpowered studies) and the extent to which the studies are compatible (in terms of populations controls and interventions) and therefore capable of being combined.

Where meta-analysis was considered appropriate authors should have explained their decisions to use fixed or random effects models in the case of RCTs, and set out the methods they intended to use to investigate heterogeneity.

With NRSI study populations vary greatly in size from small cohorts (of tens or hundreds of participants) to studies of hundreds of thousands of individuals and thousands of events. If these results, are going to be combined with those from smaller RCTs the pooled estimates of effect will be dominated by the data from the non-randomized studies. In addition, the results from NRSI may be affected by a range of biases (see above), meaning that the overall pooled estimates may be precise but biased.

Review authors should report pooled estimates separately for the different study types. In the case of NRSI, pooling may result in a very precise and 'statistically significant', but biased, estimate of effect. However, the confidence interval is calculated on the assumption that there is no bias (i.e. the estimates are as accurate as if obtained from a high quality RCT with the same number of participants). It is rare for a NRSI to have as low risk of bias as a high quality RCT of the same research question and confidence intervals for NRSI (and pooled estimates based on NRSI) should be viewed with caution. This issue is important when considering the varying risk of bias, and uncertainty about the risk of bias across NRSI.

Heterogeneity is an important issue in any meta-analysis. It is particularly important in a review of NRSI because of the more diverse methods that are likely to have been used across different studies. In addition to the usual sources of heterogeneity [different comparators, variations in baseline risk of outcomes or other characteristics of the study population, differing interventions (e.g. dose effects, context/setting, practitioner experience) and different definitions of outcomes], it is important to consider heterogeneity in source of participants, completeness of data, methods of data management and analysis. Statistical adjustment of intervention effects for confounders may result in estimates that are quite different from the unadjusted estimate derived from the raw data.

Generally, when combining the results of NRSI review authors should pool the fully adjusted estimates of effect, not the raw data. If they do the latter there should be a clear justification. However, different studies are very likely to report treatment effects that have been adjusted for different sets of covariates (or covariates measured or fitted in different ways); this diversity represents another source of potential heterogeneity.

Item 12: If meta-analysis was performed did the review authors assess the potential impact of RoB in individual studies on the results of the meta-analysis or other evidence synthesis?

In cases where review authors have chosen to include only high quality RCTs there may be little discussion of the potential impact of bias on the results. But where they have included RCTs of variable quality they should assess the impact of this by regression analysis, or by estimating pooled effect sizes with only studies at low ROB. In the case of NRSI they should estimate pooled effect sizes while including only studies at low or moderate risk of bias, and/or only those at low ROB (if there are any). If meta-analyses (or other data synthesis techniques such as regression analysis) were not performed the authors should still provide some commentary on the likely impact of ROB on individual study results.

Item 13: Did the review authors account for RoB in individual studies when interpreting/ discussing the results of the review?

Even if meta-analyses were not conducted review authors should include discussion of the impact of ROB in the interpretation of the results of the review. This is always important, but especially when reviews include RCTs with variable ROB, and with any review that includes NRSI. This discussion should not be limited to the impact of ROB on the pooled estimates (see above), but should also consider whether it may account for differences between the results of individual studies. The authors should make an explicit consideration of ROB if they make any recommendations that are likely to have an impact on clinical care or policy.

Item 14: Did the review authors provide a satisfactory explanation for, and discussion of, any heterogeneity observed in the results of the review?

There are many potential causes of heterogeneity in the results of NRSI than in RCTs. Many factors considered in this instrument, including different study designs, different methods of analysis, different populations and differing intensities of the intervention(s) – dosages in the case of drugs. Both the PICO elements and the domains of bias listed in Item 9 should also be considered as important potential sources of heterogeneity in the results. Review authors should explore these possibilities and discuss the impact of heterogeneity on the results conclusions and any recommendations

Item 15: If they performed quantitative synthesis did the review authors carry out an adequate investigation of publication bias (small study bias) and discuss its likely impact on the results of the review?

This is a very important issue, but can be difficult for review authors and appraisers to resolve completely. Typically, statistical tests or graphical displays are used and if they are positive then it indicates the presence of PB. However, negative tests are not a guarantee of the absence of PB as the tests are insensitive. To some extent the importance of PB depends on context and setting. For instance, a series of apparently methodologically sound industry-sponsored studies (e.g.

drugs, devices, putative toxins) might be more likely to be affected by PB than similar studies conducted independently of industry. The key issues are whether the authors have done their best to identify PB through deeper and intensive literature searches (as needed and according to the setting), shown an awareness of the likely impact of PB in their interpretation and discussion of the results and performed a sensitivity analyses to determine how many missing 'null' studies would be needed to invalidate the results they obtained.

Item 16: Did the review authors report any potential sources of conflict of interest, including any funding they received for conducting the review?

As noted above (under ROB), individual studies funded by vested interests may generate results that are more likely to favour the intervention than do independent studies. The same assumption applies to systematic reviews and authors should report their direct funding sources. Journals generally will require this. But assessment of the reviewers' conflicts of interest doesn't stop there. They should report their other ties. The review may be independently funded, but the authors have ties to companies that manufacture products included in the systematic review. Professional conflicts of interest are powerful, but harder to discern as they are seldom reported. When investigators have a career-long investment in a field of research, a review that conflicts with their long-held beliefs can be confronting. Potential conflicts of interest of this type will be hard to assess, but may be inferred from the fact that the reviewers have published extensively in the field being reviewed and their studies are included in the systematic review. While it can be argued that the effects of competing interests might manifest as flaws in the other domains of bias we believe that this item should always be rated separately.

To cite: Shea BJ, Reeves BC, Wells G, Thuku M, Hamel C, Moran J, Moher D, Tugwell P, Welch V, Kristjansson E, Henry DA. AMSTAR 2: a critical appraisal tool for systematic reviews that include randomised or non-randomised studies of healthcare interventions, or both. BMJ. 2017 Sep 21;358:j4008.
