# Supplementary material for: A Systematic Umbrella Review of the Effects of Teledentistry on Costs and Oral-Health Outcomes
Source: Int J Environ Res Public Health. 2024 Mar 28;21(4):407. doi: 10.3390/ijerph21040407 (PMC11050059; doi:10.3390/ijerph21040407)
Supplement: Supplementary file 1 [file ijerph-21-00407-s001.zip › ijerph-2829040-supplementary.pdf]

Table S1: Search strategies for all consulted databases

Database searched	Platform	Years of coverage	Records	Records after duplicates removed
PubMed	PubMed	1946 – Present	386	386
Embase	Embase.com	1971 - Present	465	270
Cinahl	EBSCO	1982 - Present	123	63
Web of Science Core Collection*	Clarivate	1900 - Present	156	45
Scopus	Elsevier	1970 - Present	234	30
Cochrane Database of Systematic Reviews	Wiley	1992 - Present	63	62
NHS EED + HTA	University of York	1960 - Present	24	23
JBI	Joanna Briggs Institute	2012 - Present	1	1
**Total**			1452	**880**

	**PubMed from 1946 to April 24th 2023**	
Search	Query	Results
#6	#4 AND #5	386
#5	("Meta-Analysis" [Publication Type] OR "Review"[Publication Type:NoExp] OR "Meta-Analysis as Topic"[Mesh] OR "Systematic Review" [Publication Type] OR "Cochrane Database Syst Rev"[Journal] OR metaanaly*[tiab] OR meta-analy*[tiab] or metanaly*[tiab] OR prisma[tiab] OR "preferred reporting items"[tiab] OR prospero[tiab] OR ((systemati*[ti] OR umbrella[ti] OR "structured literature"[ti]) AND (review[ti] OR overview[ti])) OR "systematic review"[tiab] OR "umbrella review"[tiab] OR "structured literature review"[tiab] OR "systematic qualitative review"[tiab] OR "systematic quantitative review"[tiab] OR "systematic search and review"[tiab] OR "systematized review"[tiab] OR "systematised review"[tiab] OR "systemic review"[tiab] OR "systematic literature review"[tiab] OR "systematic integrative literature review"[tiab] OR "systematically review"[tiab] OR "scoping literature review"[tiab] OR "scoping review"[tiab] OR "systematic critical review"[tiab] OR "systematic integrative review"[tiab] OR "systematic evidence review"[tiab] OR "systematic integrative literature review"[tiab] OR "systematic mixed studies review"[tiab] OR "systematized literature review"[tiab] OR "systematic overview"[tiab] OR "Systematic narrative review"[tiab] OR "narrative review"[tiab] OR metasynthes*[tiab] OR meta-synthes*[tiab]) NOT ("Comment" [Publication Type] OR "Letter" [Publication Type] OR "Editorial" [Publication Type] OR (("Animals"[Mesh] OR "Models, Animal"[Mesh]) NOT "Humans"[Mesh]))	3,177,383
#4	(#1 AND #3) OR #2	4,620
#3	"Telemedicine"[Mesh] OR "Remote consultation"[Mesh] OR "Distance Counseling"[Mesh] OR "Telecommunications"[Mesh] OR "Internet-based intervention"[Mesh] OR "Telenursing"[Mesh] OR "Digital technology"[Mesh] OR "Mobile applications"[Mesh] OR "Text-messaging"[Mesh] OR "Cell phone"[Mesh] OR "Smartphone"[Mesh] OR "Electronic Health Records"[Mesh] OR "Videoconferencing"[MeSH] OR "Teleradiology"[MeSH] OR "Telepathology"[MeSH] OR "mobile health"[tiab] OR Telehealth*[tiab] OR "m-health"[tiab] OR "e-health"[tiab] OR ehealth[tiab] OR mhealth*[tiab] OR telemedicine[tiab] OR "tele-medicine"[tiab] OR telepsychiatr*[tiab] OR "tele-psychiatr*"[tiab] OR telepsychotherap*[tiab] OR "tele-psychotherap*"[tiab] OR "tele-health*"[tiab] OR "Remote consult*"[tiab] OR teleconsult*[tiab] OR "distance counseling"[tiab] OR "e-therap*"[tiab] OR "e-counseling"[tiab] OR Telecommunication*[tiab] OR teleconferenc*[tiab] OR	272,933

	PubMed from 1946 to April 24th 2023	
Search	Query	Results
	"tele-conferenc*"[tiab] OR "digital health*"[tiab] OR telerehabilitation[tiab] OR "tele-rehabilitation"[tiab] OR "virtual rehabilitation"[tiab] OR "e rehabilitation"[tiab] OR erehabilitation[tiab] OR "remote rehabilitation"[tiab] OR teleexercise*[tiab] OR "tele exercise*"[tiab] OR telenutrition[tiab] OR "tele nutrition"[tiab] OR "e nutrition"[tiab] OR enutrition[tiab] OR telenutrition[tiab] OR "tele nutrition"[tiab] OR "e therap*"[tiab] OR etherap*[tiab] OR "mobile tool*"[tiab] OR "touch screen*"[tiab] OR "remote health care"[tiab] OR "remote healthcare"[tiab] OR "remote deliver*"[tiab] OR "mobile technolog*"[tiab] OR "computer intervention*"[tiab] OR "internet based"[tiab] OR "remote monitor*"[tiab] OR "tele consult*"[tiab] OR teleconsult*[tiab] OR "tele practice*"[tiab] OR landline[tiab] OR landlines[tiab] OR skype[tiab] OR facetime[tiab] OR "tablet device*"[tiab] OR "mobile app"[tiab] OR "mobile apps"[tiab] OR "mobile application*"[tiab] OR "palm pilot*"[tiab] OR pager[tiab] OR pagers[tiab] OR "mobile device"[tiab] OR "mobile devices"[tiab] OR "mobile phone"[tiab] OR "mobile phones"[tiab] OR "cellular phone"[tiab] OR "cellular phones"[tiab] OR "cellular device"[tiab] OR "cellular devices"[tiab] OR "tablet computer"[tiab] OR "tablet computers"[tiab] OR blackberry[tiab] OR android[tiab] OR laptop[tiab] OR laptops[tiab] OR "tablet personal computer"[tiab] OR "tablet personal computers"[tiab] OR Wii[tiab] OR "store and forward"[tiab] OR videoconference[tiab] OR videoconferences[tiab] OR videoconferencing[tiab] OR "video conference"[tiab] OR "video conferencing"[tiab] OR "video conferences"[tiab] OR "video consultation"[tiab] OR "video consultations"[tiab] OR wireless[tiab] OR telemetry[tiab] OR telemetric[tiab] OR telemetrical[tiab] OR biotelemetry[tiab] OR biotelemetric[tiab] OR biotelemetrical[tiab] OR bluetooth[tiab] OR "smart device"[tiab] OR "smart devices"[tiab] OR "automated reminder"[tiab] OR "automated reminders"[tiab] OR "synchronous video"[tiab] OR "live video"[tiab] OR "live videos"[tiab] OR "remote patient monitoring"[tiab] OR "home monitor"[tiab] OR "home monitors"[tiab] OR "home monitoring"[tiab] OR telerounding[tiab] OR teleround[tiab] OR telerounds[tiab] OR "tele rounds"[tiab] OR "tele rounding"[tiab] OR telehomecare[tiab] OR telehome[tiab] OR "tele home care"[tiab] OR "tele homecare"[tiab] OR "tele home health"[tiab] OR televisit[tiab] OR televisits[tiab] OR "tele visit"[tiab] OR "tele visits"[tiab] OR teleradiology[tiab] OR "tele radiology"[tiab] OR teledermatology[tiab] OR "tele dermatology"[tiab] OR teleneurology[tiab] OR "tele neurology"[tiab] OR telepharmacy[tiab] OR "tele pharmacy"[tiab] OR "telemental health"[tiab] OR "tele mental health"[tiab] OR "tele behavioral"[tiab] OR teleophthalmology[tiab] OR "tele ophthalmology"[tiab] OR telepathology[tiab] OR "tele pathology"[tiab] OR telediagnosis[tiab] OR "tele diagnosis"[tiab] OR telediagnoses[tiab] OR telementor[tiab] OR telementoring[tiab] OR telementors[tiab] OR "tele mentor"[tiab] OR "tele mentoring"[tiab] OR "virtual healthcare"[tiab] OR "virtual health care"[tiab] OR "digital health care"[tiab] OR "digital healthcare"[tiab] OR telepresence[tiab] OR evisit[tiab] OR evisits[tiab] OR "e visit"[tiab] OR "e visits"[tiab] OR eICU[tiab] OR "e ICU"[tiab] OR teledentistry[tiab] OR "remote screening"[tiab] OR "internet-based intervention*"[tiab] OR "web-based intervention*"[tiab] OR "online intervention*"[tiab] OR "tele-triage"[tiab] OR telemonitoring[tiab] OR "tele-monitoring"[tiab] OR telediagnos*[tiab] OR "tele-diagnos*"[tiab] OR telecare[tiab] OR "tele-care"[tiab] OR "virtual care"[tiab] OR teleprevent*[tiab] OR "e-prescription"[tiab] OR Gamification[tiab] OR ghealth[tiab] OR "serious gam*"[tiab] OR "Digital technolog*"[tiab] OR "Digital electronic*"[tiab] OR "mobile technolog*"[tiab] OR "Portable Electronic App*"[tiab] OR "Portable Software App*"[tiab] OR "text-messag*"[tiab] OR "texting"[tiab] OR "Short message service"[tiab] OR "Cell phone*"[tiab] OR smartphone*[tiab] OR "smart phone*"[tiab] OR "i-Phone*"[tiab] OR "i-Pad"[tiab] OR (("intra-oral camera*"[tiab] OR "oral camera*"[tiab] OR "real-time*"[tiab] OR "store-and-forward"[tiab]) AND (remote[tiab] OR distan*[tiab]))	
#2	teledent*[tiab] OR "tele-dent*"[tiab]	393
#1	"Dentistry"[Mesh] OR "Oral Health"[Mesh] OR "Stomatognathic Diseases"[Mesh] OR "Dental Clinics"[Mesh] OR "Stomatognathic Disease*"[tiab] OR "Mouth and Tooth Disease*"[tiab] OR "Dental Disease*"[tiab] OR dentist*[tiab] OR dental[tiab] OR caries[tiab] OR periodont*[tiab] OR "dental care"[tiab] OR "mouth care"[tiab] OR "dental hygiene"[tiab] OR "dental health services"[tiab] OR "oral health*"[tiab] OR "oral hygiene*"[tiab] OR "oral medicine*"[tiab] OR "mouth disease*"[tiab] OR "oral healthcare"[tiab] OR "oral health care"[tiab] OR "oral cancer*"[tiab] OR "oral neoplasm*"[tiab] OR "mouth cancer*"[tiab] OR "oral leukoplakia"[tiab] OR "lip cancer*"[tiab] OR "lip neoplasm*"[tiab] OR "tongue cancer*"[tiab] OR "tongue neoplasm*"[tiab] OR "salivary gland cancer*"[tiab] OR "salivary gland neoplasm*"[tiab] OR "oral lesion*"[tiab] OR "oral ulcer*"[tiab] OR "mouth ulcer*"[tiab] OR "buccal ulcer*"[tiab] OR "bacterial oral	1,021,159

	PubMed from 1946 to April 24th 2023	
Search	Query	Results
	infection*"[tiab] OR Noma[tiab] OR "fungal oral infection*"[tiab] OR stomatitis[tiab] OR "oral mucositis"[tiab] OR "tooth decay"[tiab] OR "tooth fissure*"[tiab] OR "dental fissure*"[tiab] OR "tooth disease*"[tiab] OR "tooth loss*"[tiab] OR "teeth loss*"[tiab] OR edentulism[tiab] OR edentulous[tiab] OR toothless[tiab] OR "lacking teeth"[tiab] OR xerostomia[tiab] OR hyposalivation[tiab] OR "mouth dryness"[tiab] OR "oral dryness"[tiab] OR "oral health education"[tiab] OR "oral health information"[tiab] OR "oral health literacy"[tiab] OR "oral health hygiene"[tiab] OR toothbrush*[tiab] OR "tooth brush*"[tiab] OR flossing[tiab] OR "oral pain"[tiab] OR "dental pain*"[tiab] OR "mouth pain"[tiab] OR gingivitis[tiab] OR "tooth wear"[tiab] OR "tooth erosion*"[tiab] OR "tooth abrasion*"[tiab] OR "dental abrasion*"[tiab] OR "dental erosion*"[tiab] OR "tooth abscess*"[tiab] OR "oral abscess*"[tiab] OR "dental abscess*"[tiab] OR "oral cyst*"[tiab] OR "access to oral care"[tiab] OR "access to dental care"[tiab]	

Key: [Mesh]= medical subject heading, [tiab] = title, abstract, author supplied keywords.

	Embase.com from inception to April 24th 2023	
No.	Query	Results
#7	#6 AND ('article'/it OR 'article in press'/it OR 'conference paper'/it OR 'conference review'/it OR 'editorial'/it OR 'letter'/it OR 'note'/it OR 'review'/it OR 'short survey'/it)	465
#6	#4 AND #5	482
#5	'meta analysis'/exp OR 'review'/exp OR 'meta analysis (topic)'/exp OR 'systematic review (topic)'/exp OR 'cochrane database of systematic reviews'/jt OR metaanaly*:ab,ti,kw OR 'meta analy*':ab,ti,kw OR metanaly*:ab,ti,kw OR prisma:ab,ti,kw OR 'preferred reporting items':ab,ti,kw OR prospero:ab,ti,kw OR (((systemati* OR umbrella OR 'structured literature') NEAR/4 (review OR overview)):ti) OR 'systematic review':ab,ti,kw OR 'umbrella review':ab,ti,kw OR 'structured literature review':ab,ti,kw OR 'systematic qualitative review':ab,ti,kw OR 'systematic quantitative review':ab,ti,kw OR 'systematic search and review':ab,ti,kw OR 'systematized review':ab,ti,kw OR 'systematised review':ab,ti,kw OR 'systemic review':ab,ti,kw OR 'systematic literature review':ab,ti,kw OR 'systematically review':ab,ti,kw OR 'scoping literature review':ab,ti,kw OR 'scoping review':ab,ti,kw OR 'systematic critical review':ab,ti,kw OR 'systematic integrative review':ab,ti,kw OR 'systematic evidence review':ab,ti,kw OR 'systematic integrative literature review':ab,ti,kw OR 'systematic mixed studies review':ab,ti,kw OR 'systematized literature review':ab,ti,kw OR 'systematic overview':ab,ti,kw OR 'systematic narrative review':ab,ti,kw OR 'narrative review':ab,ti,kw OR metasynthes*:ab,ti,kw OR 'meta synthes*':ab,ti,kw	3,388,590
#4	#1 AND #3 OR #2	4,692
#3	'telehealth'/exp OR 'e-counseling'/exp OR 'telecommunication'/exp OR 'web-based intervention'/exp OR 'digital technology'/exp OR 'mobile application'/exp OR 'text messaging'/exp OR 'mobile phone'/exp OR 'electronic health record'/exp OR 'videoconferencing'/exp OR 'mobile health':ab,ti,kw OR telehealth*:ab,ti,kw OR 'm-health':ab,ti,kw OR 'e-health':ab,ti,kw OR ehealth:ab,ti,kw OR mhealth*:ab,ti,kw OR 'telemedicine':ab,ti,kw OR 'tele-medicine':ab,ti,kw OR telepsychiatr*:ab,ti,kw OR 'tele-psychiatr*':ab,ti,kw OR telepsychotherap*:ab,ti,kw OR 'tele-psychotherap*':ab,ti,kw OR 'tele-health*':ab,ti,kw OR 'remote consult*':ab,ti,kw OR 'distance counseling':ab,ti,kw OR 'e-therap*':ab,ti,kw OR 'e-counseling':ab,ti,kw	326,507

	OR telecommunication*:ab,ti,kw OR teleconferenc*:ab,ti,kw OR 'tele-conferenc*':ab,ti,kw OR 'digital health*':ab,ti,kw OR telerehabilitation:ab,ti,kw OR 'tele-rehabilitation':ab,ti,kw OR 'virtual rehabilitation':ab,ti,kw OR 'e rehabilitation':ab,ti,kw OR erehabilitation:ab,ti,kw OR 'remote rehabilitation':ab,ti,kw OR teleexercise*:ab,ti,kw OR 'tele exercise*':ab,ti,kw OR etherap*:ab,ti,kw OR 'e nutrition':ab,ti,kw OR enutrition:ab,ti,kw OR telenutrition:ab,ti,kw OR 'tele nutrition':ab,ti,kw OR 'mobile tool*':ab,ti,kw OR 'touch screen*':ab,ti,kw OR 'remote health care':ab,ti,kw OR 'remote healthcare':ab,ti,kw OR 'remote consultation*':ab,ti,kw OR 'remote deliver*':ab,ti,kw OR 'computer intervention*':ab,ti,kw OR 'internet based':ab,ti,kw OR 'remote monitor*':ab,ti,kw OR 'tele consult*':ab,ti,kw OR teleconsult*:ab,ti,kw OR 'tele practice*':ab,ti,kw OR landline:ab,ti,kw OR landlines:ab,ti,kw OR skype:ab,ti,kw OR facetime:ab,ti,kw OR 'tablet device*':ab,ti,kw OR 'mobile app':ab,ti,kw OR 'mobile apps':ab,ti,kw OR 'mobile application*':ab,ti,kw OR 'palm pilot*':ab,ti,kw OR pager:ab,ti,kw OR pagers:ab,ti,kw OR 'mobile device':ab,ti,kw OR 'mobile devices':ab,ti,kw OR 'mobile phone':ab,ti,kw OR 'mobile phones':ab,ti,kw OR 'cellular phone':ab,ti,kw OR 'cellular phones':ab,ti,kw OR 'cellular device':ab,ti,kw OR 'cellular devices':ab,ti,kw OR 'tablet computer':ab,ti,kw OR 'tablet computers':ab,ti,kw OR blackberry:ab,ti,kw OR android:ab,ti,kw OR laptop:ab,ti,kw OR laptops:ab,ti,kw OR 'tablet personal computer':ab,ti,kw OR 'tablet personal computers':ab,ti,kw OR wii:ab,ti,kw OR 'store and forward':ab,ti,kw OR videoconference:ab,ti,kw OR videoconferences:ab,ti,kw OR videoconferencing:ab,ti,kw OR 'video conference':ab,ti,kw OR 'video conferencing':ab,ti,kw OR 'video conferences':ab,ti,kw OR 'video consultation':ab,ti,kw OR 'video consultations':ab,ti,kw OR wireless:ab,ti,kw OR telemetry:ab,ti,kw OR telemetric:ab,ti,kw OR telemetrical:ab,ti,kw OR biotelemetry:ab,ti,kw OR biotelemetric:ab,ti,kw OR biotelemetrical:ab,ti,kw OR bluetooth:ab,ti,kw OR 'smart device':ab,ti,kw OR 'smart devices':ab,ti,kw OR 'automated reminder':ab,ti,kw OR 'automated reminders':ab,ti,kw OR 'synchronous video':ab,ti,kw OR 'live video':ab,ti,kw OR 'live videos':ab,ti,kw OR 'remote patient monitoring':ab,ti,kw OR 'home monitor':ab,ti,kw OR 'home monitors':ab,ti,kw OR 'home monitoring':ab,ti,kw OR telerounding:ab,ti,kw OR teleround:ab,ti,kw OR telerounds:ab,ti,kw OR 'tele rounds':ab,ti,kw OR 'tele rounding':ab,ti,kw OR telehomecare:ab,ti,kw OR telehome:ab,ti,kw OR 'tele home care':ab,ti,kw OR 'tele homecare':ab,ti,kw OR 'tele home health':ab,ti,kw OR televisit:ab,ti,kw OR televisits:ab,ti,kw OR 'tele visit':ab,ti,kw OR 'tele visits':ab,ti,kw OR teleradiology:ab,ti,kw OR 'tele radiology':ab,ti,kw OR teledermatology:ab,ti,kw OR 'tele dermatology':ab,ti,kw OR teleneurology:ab,ti,kw OR 'tele neurology':ab,ti,kw OR telepharmacy:ab,ti,kw OR 'tele pharmacy':ab,ti,kw OR 'telemental health':ab,ti,kw OR 'tele mental health':ab,ti,kw OR 'tele behavioral':ab,ti,kw OR teleophthalmology:ab,ti,kw OR 'tele ophthalmology':ab,ti,kw OR telepathology:ab,ti,kw OR 'tele pathology':ab,ti,kw OR telediagnosis:ab,ti,kw OR 'tele diagnosis':ab,ti,kw OR telediagnoses:ab,ti,kw OR telementor:ab,ti,kw OR telementoring:ab,ti,kw OR telementors:ab,ti,kw OR 'tele mentor':ab,ti,kw OR 'tele mentoring':ab,ti,kw OR 'virtual healthcare':ab,ti,kw OR 'virtual health care':ab,ti,kw OR 'digital health care':ab,ti,kw OR 'digital healthcare':ab,ti,kw OR telepresence:ab,ti,kw OR evisit:ab,ti,kw OR evisits:ab,ti,kw OR 'e visit':ab,ti,kw OR 'e visits':ab,ti,kw OR eicu:ab,ti,kw OR 'e icu':ab,ti,kw OR teledentistry:ab,ti,kw OR 'remote screening':ab,ti,kw OR 'internet-based intervention*':ab,ti,kw OR 'web-based intervention*':ab,ti,kw OR 'online intervention*':ab,ti,kw OR 'tele-triage':ab,ti,kw OR 'telemonitoring':ab,ti,kw OR 'tele-monitoring':ab,ti,kw OR telediagnos*:ab,ti,kw	

	OR 'tele-diagnos*':ab,ti,kw OR telecare:ab,ti,kw OR 'tele-care':ab,ti,kw OR 'virtual care':ab,ti,kw OR teleprevent*:ab,ti,kw OR 'e-prescription':ab,ti,kw OR gamification:ab,ti,kw OR ghealth:ab,ti,kw OR 'serious gam*':ab,ti,kw OR 'digital technolog*':ab,ti,kw OR 'digital electronic*':ab,ti,kw OR 'mobile technolog*':ab,ti,kw OR 'portable electronic app*':ab,ti,kw OR 'portable software app*':ab,ti,kw OR 'text-messag*':ab,ti,kw OR 'texting':ab,ti,kw OR 'short message service':ab,ti,kw OR 'cell phone*':ab,ti,kw OR smartphone*:ab,ti,kw OR 'smart phone*':ab,ti,kw OR 'i-phone*':ab,ti,kw OR 'i-pad':ab,ti,kw OR ((('intra-oral camera*' OR 'oral camera*' OR 'real-time*' OR 'store-and-forward') NEAR/4 (remote OR distan*)):ab,ti,kw)	
#2	'teledentistry'/exp OR teledent*:ab,ti,kw OR 'tele-dent*':ab,ti,kw	424
#1	'dentistry'/exp OR 'mouth disease'/exp OR 'dental clinic'/exp OR 'stomatognathic disease*':ab,ti,kw OR 'mouth and tooth disease*':ab,ti,kw OR 'dental disease*':ab,ti,kw OR dentist*:ab,ti,kw OR dental:ab,ti,kw OR caries:ab,ti,kw OR periodont*:ab,ti,kw OR 'dental care':ab,ti,kw OR 'mouth care':ab,ti,kw OR 'dental hygiene':ab,ti,kw OR 'dental health services':ab,ti,kw OR 'oral health*':ab,ti,kw OR 'oral hygiene*':ab,ti,kw OR 'oral medicine*':ab,ti,kw OR 'mouth disease*':ab,ti,kw OR 'oral healthcare':ab,ti,kw OR 'oral health care':ab,ti,kw OR 'oral cancer*':ab,ti,kw OR 'oral neoplasm*':ab,ti,kw OR 'mouth cancer*':ab,ti,kw OR 'oral leukoplakia':ab,ti,kw OR 'lip cancer*':ab,ti,kw OR 'lip neoplasm*':ab,ti,kw OR 'tongue cancer*':ab,ti,kw OR 'tongue neoplasm*':ab,ti,kw OR 'salivary gland cancer*':ab,ti,kw OR 'salivary gland neoplasm*':ab,ti,kw OR 'oral lesion*':ab,ti,kw OR 'oral ulcer*':ab,ti,kw OR 'mouth ulcer*':ab,ti,kw OR 'buccal ulcer*':ab,ti,kw OR 'bacterial oral infection*':ab,ti,kw OR noma:ab,ti,kw OR 'fungal oral infection*':ab,ti,kw OR stomatitis:ab,ti,kw OR 'oral mucositis':ab,ti,kw OR 'tooth decay':ab,ti,kw OR 'tooth fissure*':ab,ti,kw OR 'dental fissure*':ab,ti,kw OR 'tooth disease*':ab,ti,kw OR 'tooth loss*':ab,ti,kw OR 'teeth loss*':ab,ti,kw OR edentulism:ab,ti,kw OR edentulous:ab,ti,kw OR toothless:ab,ti,kw OR 'lacking teeth':ab,ti,kw OR xerostomia:ab,ti,kw OR hyposalivation:ab,ti,kw OR 'mouth dryness':ab,ti,kw OR 'oral dryness':ab,ti,kw OR 'oral health education':ab,ti,kw OR 'oral health information':ab,ti,kw OR 'oral health literacy':ab,ti,kw OR 'oral health hygiene':ab,ti,kw OR toothbrush*:ab,ti,kw OR 'tooth brush*':ab,ti,kw OR flossing:ab,ti,kw OR 'oral pain':ab,ti,kw OR 'dental pain*':ab,ti,kw OR 'mouth pain':ab,ti,kw OR gingivitis:ab,ti,kw OR 'tooth wear':ab,ti,kw OR 'tooth erosion*':ab,ti,kw OR 'tooth abrasion*':ab,ti,kw OR 'dental abrasion*':ab,ti,kw OR 'dental erosion*':ab,ti,kw OR 'tooth abscess*':ab,ti,kw OR 'oral abscess*':ab,ti,kw OR 'dental abscess*':ab,ti,kw OR 'oral cyst*':ab,ti,kw OR 'access to oral care':ab,ti,kw OR 'access to dental care':ab,ti,kw	1,038,999

Key: Ab, ti, kw searches in abstract, title and author supplied keywords, /exp searches Emtree preferred indexing term, it=publication type

	**Cinahl (EBSCO) from inception to April 24th 2023**		
**#**	**Query**	**Limiters/Expanders**	**Results**
#7	#4 AND #5	Limiters –	123

		Academic journals	
#6	#4 AND #5	Search modes - Boolean/Phrase	126
#5	(MH "Meta Analysis") OR (MH "Scoping Review") OR (MH "Systematic Review") OR TI(metaanaly* OR meta-analy* or metanaly* OR prisma OR "preferred reporting items" OR prospero OR ((systemati* OR umbrella OR "structured literature") W4 (review OR overview)) OR "systematic review" OR "umbrella review" OR "structured literature review" OR "systematic qualitative review" OR "systematic quantitative review" OR "systematic search and review" OR "systematized review" OR "systematised review" OR "systemic review" OR "systematic literature review" OR "systematic integrative literature review" OR "systematically review" OR "scoping literature review" OR "scoping review" OR "systematic critical review" OR "systematic integrative review" OR "systematic evidence review" OR "systematic integrative literature review" OR "systematic mixed studies review" OR "systematized literature review" OR "systematic overview" OR "Systematic narrative review" OR "narrative review" OR metasynthes* OR meta-synthes*) OR AB(metaanaly* OR meta-analy* or metanaly* OR prisma OR "preferred reporting items" OR prospero OR "systematic review" OR "umbrella review" OR "structured literature review" OR "systematic qualitative review" OR "systematic quantitative review" OR "systematic search and review" OR "systematized review" OR "systematised review" OR "systemic review" OR "systematic literature review" OR "systematic integrative literature review" OR "systematically review" OR "scoping literature review" OR "scoping review" OR "systematic critical review" OR "systematic integrative review" OR "systematic evidence review" OR "systematic integrative literature review" OR "systematic mixed studies review" OR "systematized literature review" OR "systematic overview" OR "Systematic narrative review" OR "narrative review" OR metasynthes* OR meta-synthes*) OR SU(metaanaly* OR meta-analy* or metanaly* OR prisma OR "preferred reporting items" OR prospero OR "systematic review" OR "umbrella review" OR "structured literature review" OR "systematic qualitative review" OR "systematic quantitative review" OR "systematic search and review" OR "systematized review" OR "systematised review" OR "systemic review" OR "systematic literature review" OR "systematic integrative literature review" OR "systematically review" OR "scoping literature review" OR "scoping review" OR "systematic critical review" OR "systematic integrative review" OR "systematic evidence review" OR "systematic integrative literature review" OR "systematic mixed studies review" OR "systematized literature review" OR "systematic overview" OR "Systematic narrative review" OR "narrative review" OR metasynthes* OR meta-synthes*)	Search modes - Boolean/Phrase	244,231
#4	(#1 AND #3) OR #2	Search modes - Boolean/Phrase	5,332

#3	(MH "Telehealth+") OR (MH "Telemedicine+") OR (MM "Remote Consultation") OR (MH "Telecommunications+") OR (MM "Internet-Based Intervention") OR (MH "Digital Technology+") OR (MM "Mobile Applications") OR (MH "Text Messaging") OR (MH "Cellular Phone") OR (MH "Smartphone") OR (MH "Electronic Health Records+") OR (MH "Electronic Health Records+") OR TI("mobile health" OR Telehealth* OR "m-health" OR "e-health" OR ehealth OR mhealth* OR telemedicine OR "tele-medicine" OR telepsychiatr* OR "tele-psychiatr*" OR telepsychotherap* OR "tele-psychotherap*" OR "tele-health*" OR "Remote consult*" OR teleconsult* OR "distance counseling" OR "e-therap*" OR "e-counseling" OR Telecommunication* OR teleconferenc* OR "tele-conferenc*" OR "digital health*" OR telerehabilitation OR "tele-rehabilitation" OR "virtual rehabilitation" OR "e rehabilitation" OR erehabilitation OR "remote rehabilitation" OR teleexercise* OR "tele exercise*" OR telenutrition OR "tele nutrition" OR "e nutrition" OR enutrition OR telenutrition OR "tele nutrition" OR etherap* OR "mobile tool*" OR "touch screen*" OR "remote health care" OR "remote healthcare" OR "remote deliver*" OR "mobile technolog*" OR "computer intervention*" OR "internet based" OR "remote monitor*" OR "tele consult*" OR teleconsult* OR "tele practice*" OR landline OR landlines OR skype OR facetime OR "tablet device*" OR "mobile app" OR "mobile apps" OR "mobile application*" OR "palm pilot*" OR pager OR pagers OR "mobile device" OR "mobile devices" OR "mobile phone" OR "mobile phones" OR "cellular phone" OR "cellular phones" OR "cellular device" OR "cellular devices" OR "tablet computer" OR "tablet computers" OR blackberry OR android OR laptop OR laptops OR "tablet personal computer" OR "tablet personal computers" OR Wii OR "store and forward" OR videoconference OR videoconferences OR videoconferencing OR "video conference" OR "video conferencing" OR "video conferences" OR "video consultation" OR "video consultations" OR wireless OR telemetry OR telemetric OR telemetrical OR biotelemetry OR biotelemetric OR biotelemetrical OR bluetooth OR "smart device" OR "smart devices" OR "automated reminder" OR "automated reminders" OR "synchronous video" OR "live video" OR "live videos" OR "remote patient monitoring" OR "home monitor" OR "home monitors" OR "home monitoring" OR telerounding OR teleround OR telerounds OR "tele rounds" OR "tele rounding" OR telehomecare OR telehome OR "tele home care" OR "tele homecare" OR "tele home health" OR televisit OR televisits OR "tele visit" OR "tele visits" OR teleradiology OR "tele radiology" OR teledermatology OR "tele dermatology" OR teleneurology OR "tele neurology" OR telepharmacy OR "tele pharmacy" OR "telemental health" OR "tele mental health" OR "tele behavioral" OR teleophthalmology OR "tele ophthalmology" OR telepathology OR "tele pathology" OR telediagnosis OR "tele diagnosis" OR telediagnoses OR telementor OR telementoring OR telementors OR "tele mentor" OR "tele mentoring" OR "virtual healthcare" OR "virtual health care" OR "digital health care" OR "digital healthcare" OR telepresence OR evisit OR evisits OR "e visit" OR "e visits" OR eICU OR "e ICU" OR teledentistry OR "remote screening" OR "internet-based intervention*" OR "web-based intervention*" OR "online	Search modes - Boolean/Phrase	236,891

	intervention*" OR "tele-triage" OR telemonitoring OR "tele-monitoring" OR telediagnos* OR "tele-diagnos*" OR telecare OR "tele-care" OR "virtual care" OR teleprevent* OR "e-prescription" OR Gamification OR ghealth OR "serious gam*" OR "Digital technolog*" OR "Digital electronic*" OR "mobile technolog*" OR "Portable Electronic App*" OR "Portable Software App*" OR "text-messag*" OR texting OR "Short message service" OR "Cell phone*" OR smartphone* OR "smart phone*" OR "i-Phone*" OR "i-Pad" OR (("intra-oral camera*" OR "oral camera*" OR "real-time*" OR "store-and-forward") W4 (remote OR distan*))) OR AB("mobile health" OR Telehealth* OR "m-health" OR "e-health" OR ehealth OR mhealth* OR telemedicine OR "tele-medicine" OR telepsychiatr* OR "tele-psychiatr*" OR telepsychotherap* OR "tele-psychotherap*" OR "tele-health*" OR "Remote consult*" OR teleconsult* OR "distance counseling" OR "e-therap*" OR "e-counseling" OR Telecommunication* OR teleconferenc* OR "tele-conferenc*" OR "digital health*" OR telerehabilitation OR "tele-rehabilitation" OR "virtual rehabilitation" OR "e rehabilitation" OR erehabilitation OR "remote rehabilitation" OR teleexercise* OR "tele exercise*" OR telenutrition OR "tele nutrition" OR "e nutrition" OR enutrition OR telenutrition OR "tele nutrition" OR etherap* OR "mobile tool*" OR "touch screen*" OR "remote health care" OR "remote healthcare" OR "remote deliver*" OR "mobile technolog*" OR "computer intervention*" OR "internet based" OR "remote monitor*" OR "tele consult*" OR teleconsult* OR "tele practice*" OR landline OR landlines OR skype OR facetime OR "tablet device*" OR "mobile app" OR "mobile apps" OR "mobile application*" OR "palm pilot*" OR pager OR pagers OR "mobile device" OR "mobile devices" OR "mobile phone" OR "mobile phones" OR "cellular phone" OR "cellular phones" OR "cellular device" OR "cellular devices" OR "tablet computer" OR "tablet computers" OR blackberry OR android OR laptop OR laptops OR "tablet personal computer" OR "tablet personal computers" OR Wii OR "store and forward" OR videoconference OR videoconferences OR videoconferencing OR "video conference" OR "video conferencing" OR "video conferences" OR "video consultation" OR "video consultations" OR wireless OR telemetry OR telemetric OR telemetrical OR biotelemetry OR biotelemetric OR biotelemetrical OR bluetooth OR "smart device" OR "smart devices" OR "automated reminder" OR "automated reminders" OR "synchronous video" OR "live video" OR "live videos" OR "remote patient monitoring" OR "home monitor" OR "home monitors" OR "home monitoring" OR telerounding OR teleround OR telerounds OR "tele rounds" OR "tele rounding" OR telehomecare OR telehome OR "tele home care" OR "tele homecare" OR "tele home health" OR televisit OR televisits OR "tele visit" OR "tele visits" OR teleradiology OR "tele radiology" OR teledermatology OR "tele dermatology" OR teleneurology OR "tele neurology" OR telepharmacy OR "tele pharmacy" OR "telemental health" OR "tele mental health" OR "tele behavioral" OR teleophthalmology OR "tele ophthalmology" OR telepathology OR "tele pathology" OR telediagnosis OR "tele diagnosis" OR telediagnoses OR telementor OR telementoring OR telementors OR "tele mentor" OR "tele mentoring" OR "virtual healthcare" OR "virtual health care" OR "digital health		

	care" OR "digital healthcare" OR telepresence OR evisit OR evisits OR "e visit" OR "e visits" OR eICU OR "e ICU" OR teledentistry OR "remote screening" OR "internet-based intervention*" OR "web-based intervention*" OR "online intervention*" OR "tele-triage" OR telemonitoring OR "tele-monitoring" OR telediagnos* OR "tele-diagnos*" OR telecare OR "tele-care" OR "virtual care" OR teleprevent* OR "e-prescription" OR Gamification OR ghealth OR "serious gam*" OR "Digital technolog*" OR "Digital electronic*" OR "mobile technolog*" OR "Portable Electronic App*" OR "Portable Software App*" OR "text-messag*" OR texting OR "Short message service" OR "Cell phone*" OR smartphone* OR "smart phone*" OR "i-Phone*" OR "i-Pad" OR (("intra-oral camera*" OR "oral camera*" OR "real-time*" OR "store-and-forward") W4 (remote OR distan*))) OR SU("mobile health" OR Telehealth* OR "m-health" OR "e-health" OR ehealth OR mhealth* OR telemedicine OR "tele-medicine" OR telepsychiatr* OR "tele-psychiatr*" OR telepsychotherap* OR "tele-psychotherap*" OR "tele-health*" OR "Remote consult*" OR teleconsult* OR "distance counseling" OR "e-therap*" OR "e-counseling" OR Telecommunication* OR teleconferenc* OR "tele-conferenc*" OR "digital health*" OR telerehabilitation OR "tele-rehabilitation" OR "virtual rehabilitation" OR "e rehabilitation" OR erehabilitation OR "remote rehabilitation" OR teleexercise* OR "tele exercise*" OR telenutrition OR "tele nutrition" OR "e nutrition" OR enutrition OR telenutrition OR "tele nutrition" OR etherap* OR "mobile tool*" OR "touch screen*" OR "remote health care" OR "remote healthcare" OR "remote deliver*" OR "mobile technolog*" OR "computer intervention*" OR "internet based" OR "remote monitor*" OR "tele consult*" OR teleconsult* OR "tele practice*" OR landline OR landlines OR skype OR facetime OR "tablet device*" OR "mobile app" OR "mobile apps" OR "mobile application*" OR "palm pilot*" OR pager OR pagers OR "mobile device" OR "mobile devices" OR "mobile phone" OR "mobile phones" OR "cellular phone" OR "cellular phones" OR "cellular device" OR "cellular devices" OR "tablet computer" OR "tablet computers" OR blackberry OR android OR laptop OR laptops OR "tablet personal computer" OR "tablet personal computers" OR Wii OR "store and forward" OR videoconference OR videoconferences OR videoconferencing OR "video conference" OR "video conferencing" OR "video conferences" OR "video consultation" OR "video consultations" OR wireless OR telemetry OR telemetric OR telemetrical OR biotelemetry OR biotelemetric OR biotelemetrical OR bluetooth OR "smart device" OR "smart devices" OR "automated reminder" OR "automated reminders" OR "synchronous video" OR "live video" OR "live videos" OR "remote patient monitoring" OR "home monitor" OR "home monitors" OR "home monitoring" OR telerounding OR teleround OR telerounds OR "tele rounds" OR "tele rounding" OR telehomecare OR telehome OR "tele home care" OR "tele homecare" OR "tele home health" OR televisit OR televisits OR "tele visit" OR "tele visits" OR teleradiology OR "tele radiology" OR teledermatology OR "tele dermatology" OR teleneurology OR "tele neurology" OR telepharmacy OR "tele pharmacy" OR "telemental health" OR "tele mental health" OR "tele behavioral" OR teleophthalmology OR "tele ophthalmology" OR telepathology		

	OR "tele pathology" OR telediagnosis OR "tele diagnosis" OR telediagnoses OR telementor OR telementoring OR telementors OR "tele mentor" OR "tele mentoring" OR "virtual healthcare" OR "virtual health care" OR "digital health care" OR "digital healthcare" OR telepresence OR evisit OR evisits OR "e visit" OR "e visits" OR eICU OR "e ICU" OR teledentistry OR "remote screening" OR "internet-based intervention*" OR "web-based intervention*" OR "online intervention*" OR "tele-triage" OR telemonitoring OR "tele-monitoring" OR telediagnos* OR "tele-diagnos*" OR telecare OR "tele-care" OR "virtual care" OR teleprevent* OR "e-prescription" OR Gamification OR ghealth OR "serious gam*" OR "Digital technolog*" OR "Digital electronic*" OR "mobile technolog*" OR "Portable Electronic App*" OR "Portable Software App*" OR "text-messag*" OR texting OR "Short message service" OR "Cell phone*" OR smartphone* OR "smart phone*" OR "i-Phone*" OR "i-Pad" OR (("intra-oral camera*" OR "oral camera*" OR "real-time*" OR "store-and-forward") W4 (remote OR distan*)))		
#2	(MH "Teledentistry") OR TI(teledent* OR “tele-dent*”) OR AB(teledent* OR “tele-dent*”) OR SU(teledent* OR “tele-dent*”)	Search modes - Boolean/Phrase	267
#1	(MH "Dentistry+") OR (MH "Oral Health") OR (MH "Stomatognathic Diseases+") OR (MM "Dental Clinics") OR (MM "Dental Offices") OR TI("Stomatognathic Disease*" OR "Mouth and Tooth Disease*" OR "Dental Disease*" OR dentist* OR dental OR caries OR periodont* OR "dental care" OR "mouth care" OR "dental hygiene" OR "dental health services" OR "oral health*" OR "oral hygiene*" OR "oral medicine*" OR "mouth disease*" OR "oral healthcare" OR "oral health care" OR "oral cancer*" OR "oral neoplasm*" OR "mouth cancer*" OR "oral leukoplakia" OR "lip cancer*" OR "lip neoplasm*" OR "tongue cancer*" OR "tongue neoplasm*" OR "salivary gland cancer*" OR "salivary gland neoplasm*" OR "oral lesion*" OR "oral ulcer*" OR "mouth ulcer*" OR "buccal ulcer*" OR "bacterial oral infection*" OR Noma OR "fungal oral infection*" OR stomatitis OR "oral mucositis" OR "tooth decay" OR "tooth fissure*" OR "dental fissure*" OR "tooth disease*" OR "tooth loss*" OR "teeth loss*" OR edentulism OR edentulous OR toothless OR "lacking teeth" OR xerostomia OR hyposalivation OR "mouth dryness" OR "oral dryness" OR "oral health education" OR "oral health information" OR "oral health literacy" OR "oral health hygiene" OR toothbrush* OR "tooth brush*" OR flossing OR "oral pain" OR "dental pain*" OR "mouth pain" OR gingivitis OR "tooth wear" OR "tooth erosion*" OR "tooth abrasion*" OR "dental abrasion*" OR "dental erosion*" OR "tooth abscess*" OR "oral abscess*" OR "dental abscess*" OR "oral cyst*" OR "access to oral care" OR "access to dental care") OR AB("Stomatognathic Disease*" OR "Mouth and Tooth Disease*" OR "Dental Disease*" OR dentist* OR dental OR caries OR periodont* OR "dental care" OR "mouth care" OR "dental hygiene" OR "dental health services" OR "oral health*" OR "oral hygiene*" OR "oral medicine*" OR "mouth disease*" OR "oral healthcare" OR "oral health care" OR "oral cancer*" OR "oral neoplasm*" OR "mouth cancer*" OR "oral leukoplakia" OR "lip cancer*" OR "lip neoplasm*" OR "tongue cancer*" OR "tongue neoplasm*" OR "salivary	Search modes - Boolean/Phrase	246,641

	gland cancer*" OR "salivary gland neoplasm*" OR "oral lesion*" OR "oral ulcer*" OR "mouth ulcer*" OR "buccal ulcer*" OR "bacterial oral infection*" OR Noma OR "fungal oral infection*" OR stomatitis OR "oral mucositis" OR "tooth decay" OR "tooth fissure*" OR "dental fissure*" OR "tooth disease*" OR "tooth loss*" OR "teeth loss*" OR edentulism OR edentulous OR toothless OR "lacking teeth" OR xerostomia OR hyposalivation OR "mouth dryness" OR "oral dryness" OR "oral health education" OR "oral health information" OR "oral health literacy" OR "oral health hygiene" OR toothbrush* OR "tooth brush*" OR flossing OR "oral pain" OR "dental pain*" OR "mouth pain" OR gingivitis OR "tooth wear" OR "tooth erosion*" OR "tooth abrasion*" OR "dental abrasion*" OR "dental erosion*" OR "tooth abscess*" OR "oral abscess*" OR "dental abscess*" OR "oral cyst*" OR "access to oral care" OR "access to dental care") OR SU("Stomatognathic Disease*" OR "Mouth and Tooth Disease*" OR "Dental Disease*"OR dentist* OR dental OR caries OR periodont* OR "dental care" OR "mouth care" OR "dental hygiene" OR "dental health services" OR "oral health*" OR "oral hygiene*" OR "oral medicine*" OR "mouth disease*" OR "oral healthcare" OR "oral health care" OR "oral cancer*" OR "oral neoplasm*" OR "mouth cancer*" OR "oral leukoplakia" OR "lip cancer*" OR "lip neoplasm*" OR "tongue cancer*" OR "tongue neoplasm*" OR "salivary gland cancer*" OR "salivary gland neoplasm*" OR "oral lesion*" OR "oral ulcer*" OR "mouth ulcer*" OR "buccal ulcer*" OR "bacterial oral infection*" OR Noma OR "fungal oral infection*" OR stomatitis OR "oral mucositis" OR "tooth decay" OR "tooth fissure*" OR "dental fissure*" OR "tooth disease*" OR "tooth loss*" OR "teeth loss*" OR edentulism OR edentulous OR toothless OR "lacking teeth" OR xerostomia OR hyposalivation OR "mouth dryness" OR "oral dryness" OR "oral health education" OR "oral health information" OR "oral health literacy" OR "oral health hygiene" OR toothbrush* OR "tooth brush*" OR flossing OR "oral pain" OR "dental pain*" OR "mouth pain" OR gingivitis OR "tooth wear" OR "tooth erosion*" OR "tooth abrasion*" OR "dental abrasion*" OR "dental erosion*" OR "tooth abscess*" OR "oral abscess*" OR "dental abscess*" OR "oral cyst*" OR "access to oral care" OR "access to dental care")		

Key: MH= Medical Headings,TI= title, AB= abstract and SU= Searches for keywords in the uncontrolled content description of the document

	**Web of Science Core Collection – from 1900 to April 24th 2023**	
Set	Query	Results
#6	#4 AND #5	156
#5	TS=(metaanaly* OR meta-analy* or metanaly* OR prisma OR "preferred reporting items" OR prospero OR "systematic review" OR "umbrella review" OR "structured literature review" OR "systematic qualitative review" OR "systematic quantitative review" OR "systematic search and review" OR "systematized review" OR "systematised review" OR "systemic review" OR "systematic literature review" OR "systematic integrative literature review" OR "systematically review" OR "scoping literature review" OR "scoping review" OR "systematic critical review" OR "systematic integrative review" OR "systematic evidence review" OR "systematic integrative literature review" OR "systematic mixed studies review" OR "systematized literature review" OR "systematic overview" OR "Systematic narrative review" OR "narrative review" OR metasynthes*	735,572

	OR meta-synthes*) OR TI=((systemati* OR umbrella OR "structured literature") NEAR/4 (review OR overview))	
#4	(#1 AND #3) OR #2	4,291
#3	TS=("mobile health" OR Telehealth* OR "m-health" OR "e-health" OR ehealth OR mhealth* OR telemedicine OR "tele-medicine" OR telepsychiatr* OR "tele-psychiatr*" OR telepsychotherap* OR "tele-psychotherap*" OR "tele-health*" OR "Remote consult*" OR teleconsult* OR "distance counseling" OR "e-therap*" OR "e-counseling" OR Telecommunication* OR teleconferenc* OR "tele-conferenc*" OR "digital health*" OR telerehabilitation OR "tele-rehabilitation" OR "virtual rehabilitation" OR "e rehabilitation" OR erehabilitation OR "remote rehabilitation" OR teleexercise* OR "tele exercise*" OR telenutrition OR "tele nutrition" OR "e nutrition" OR enutrition OR telenutrition OR "tele nutrition" OR etherap* OR "mobile tool*" OR "touch screen*" OR "remote health care" OR "remote healthcare" OR "remote deliver*" OR "mobile technolog*" OR "computer intervention*" OR "internet based" OR "remote monitor*" OR "tele consult*" OR teleconsult* OR "tele practice*" OR landline OR landlines OR skype OR facetime OR "tablet device*" OR "mobile app" OR "mobile apps" OR "mobile application*" OR "palm pilot*" OR pager OR pagers OR "mobile device" OR "mobile devices" OR "mobile phone" OR "mobile phones" OR "cellular phone" OR "cellular phones" OR "cellular device" OR "cellular devices" OR "tablet computer" OR "tablet computers" OR blackberry OR android OR laptop OR laptops OR "tablet personal computer" OR "tablet personal computers" OR Wii OR "store and forward" OR videoconference OR videoconferences OR videoconferencing OR "video conference" OR "video conferencing" OR "video conferences" OR "video consultation" OR "video consultations" OR wireless OR telemetry OR telemetric OR telemetrical OR biotelemetry OR biotelemetric OR biotelemetrical OR bluetooth OR "smart device" OR "smart devices" OR "automated reminder" OR "automated reminders" OR "synchronous video" OR "live video" OR "live videos" OR "remote patient monitoring" OR "home monitor" OR "home monitors" OR "home monitoring" OR telerounding OR teleround OR telerounds OR "tele rounds" OR "tele rounding" OR telehomecare OR telehome OR "tele home care" OR "tele homecare" OR "tele home health" OR televisit OR televisits OR "tele visit" OR "tele visits" OR teleradiology OR "tele radiology" OR teledermatology OR "tele dermatology" OR teleneurology OR "tele neurology" OR telepharmacy OR "tele pharmacy" OR "telemental health" OR "tele mental health" OR "tele behavioral" OR teleophthalmology OR "tele ophthalmology" OR telepathology OR "tele pathology" OR telediagnosis OR "tele diagnosis" OR telediagnoses OR telementor OR telementoring OR telementors OR "tele mentor" OR "tele mentoring" OR "virtual healthcare" OR "virtual health care" OR "digital health care" OR "digital healthcare" OR telepresence OR evisit OR evisits OR "e visit" OR "e visits" OR eICU OR "e ICU" OR teledentistry OR "remote screening" OR "internet-based intervention*" OR "web-based intervention*" OR "online intervention*" OR "tele-triage" OR telemonitoring OR "tele-monitoring" OR telediagnos* OR "tele-diagnos*" OR telecare OR "tele-care" OR "virtual care" OR teleprevent* OR "e-prescription" OR Gamification OR ghealth OR "serious gam*" OR "Digital technolog*" OR "Digital electronic*" OR "mobile technolog*" OR "Portable Electronic App*" OR "Portable Software App*" OR "text-messag*" OR texting OR "Short message service" OR "Cell phone*" OR smartphone* OR "smart phone*" OR "i-Phone*" OR "i-Pad" OR (("intra-oral camera*" OR "oral camera*" OR "real-time*" OR "store-and-forward") NEAR/4 (remote OR distan*)))	4,382,692
#2	TS=(teledent* OR “tele-dent*”)	427
#1	TS=("Stomatognathic Disease*" OR "Mouth and Tooth Disease*" OR "Dental Disease*" OR dentist* OR dental OR caries OR periodont* OR "dental care" OR "mouth care" OR "dental hygiene" OR "dental health services" OR "oral health*" OR "oral hygiene*" OR "oral medicine*" OR "mouth disease*" OR "oral healthcare" OR "oral health care" OR "oral cancer*" OR "oral neoplasm*" OR "mouth cancer*" OR "oral leukoplakia" OR "lip cancer*" OR "lip neoplasm*" OR "tongue cancer*" OR "tongue neoplasm*" OR "salivary gland cancer*" OR "salivary gland neoplasm*" OR "oral lesion*" OR "oral ulcer*" OR "mouth ulcer*" OR "buccal ulcer*" OR "bacterial oral infection*" OR Noma OR "fungal oral infection*" OR stomatitis OR "oral mucositis" OR "tooth decay" OR "tooth fissure*" OR "dental fissure*" OR "tooth disease*" OR "tooth loss*" OR "teeth loss*" OR edentulism OR edentulous OR toothless OR "lacking teeth" OR xerostomia OR hyposalivation OR "mouth dryness" OR "oral dryness" OR "oral health education" OR "oral health information" OR "oral health literacy" OR "oral health hygiene" OR toothbrush* OR "tooth brush*" OR flossing OR "oral pain" OR "dental pain*" OR "mouth pain" OR gingivitis OR "tooth wear" OR "tooth erosion*" OR "tooth abrasion*" OR "dental abrasion*" OR "dental erosion*" OR "tooth abscess*" OR "oral abscess*" OR "dental abscess*" OR "oral cyst*" OR "access to oral care" OR "access to dental care")	435,278

Key: TS = topic, which includes title, abstract, author keywords and Web of Science Keywords Plus.

	Scopus (Elsevier) from inception to April 24th 2023	
Nr.	Query	Results
6	#4 AND #5	234

5	TITLE-ABS-KEY (metaanaly* OR meta-analy* OR metanaly* OR prisma OR "preferred reporting items" OR prospero OR "systematic review" OR "umbrella review" OR "structured literature review" OR "systematic qualitative review" OR "systematic quantitative review" OR "systematic search and review" OR "systematized review" OR "systematised review" OR "systemic review" OR "systematic literature review" OR "systematic integrative literature review" OR "systematically review" OR "scoping literature review" OR "scoping review" OR "systematic critical review" OR "systematic integrative review" OR "systematic evidence review" OR "systematic integrative literature review" OR "systematic mixed studies review" OR "systematized literature review" OR "systematic overview" OR "Systematic narrative review" OR "narrative review" OR metasynthes* OR meta-synthes*) OR TITLE ((systemati* OR umbrella OR "structured literature") W/4 (review OR overview))	725,505
4	(#1 AND #3) OR #2	8,606
3	TITLE-ABS-KEY ("mobile health" OR telehealth* OR "m-health" OR "e-health" OR ehealth OR mhealth* OR telemedicine OR "tele-medicine" OR telepsychiatr* OR "tele-psychiatr*" OR telepsychotherap* OR "tele-psychotherap*" OR "tele-health*" OR "Remote consult*" OR teleconsult* OR "distance counseling" OR "e-therap*" OR "e-counseling" OR telecommunication* OR teleconferenc* OR "tele-conferenc*" OR "digital health*" OR telerehabilitation OR "tele-rehabilitation" OR "virtual rehabilitation" OR "e rehabilitation" OR erehabilitation OR "remote rehabilitation" OR teleexercise* OR "tele exercise*" OR telenutrition OR "tele nutrition" OR "e nutrition" OR enutrition OR telenutrition OR "tele nutrition" OR etherap* OR "mobile tool*" OR "touch screen*" OR "remote health care" OR "remote healthcare" OR "remote deliver*" OR "mobile technolog*" OR "computer intervention*" OR "internet based" OR "remote monitor*" OR "tele consult*" OR teleconsult* OR "tele practice*" OR landline OR landlines OR skype OR facetime OR "tablet device*" OR "mobile app" OR "mobile apps" OR "mobile application*" OR "palm pilot*" OR pager OR pagers OR "mobile device" OR "mobile devices" OR "mobile phone" OR "mobile phones" OR "cellular phone" OR "cellular phones" OR "cellular device" OR "cellular devices" OR "tablet computer" OR "tablet computers" OR blackberry OR android OR laptop OR laptops OR "tablet personal computer" OR "tablet personal computers" OR wii OR "store and forward" OR videoconference OR videoconferences OR videoconferencing OR "video conference" OR "video conferencing" OR "video conferences" OR "video consultation" OR "video consultations" OR wireless OR telemetry OR telemetric OR telemetrical OR biotelemetry OR biotelemetric OR biotelemetrical OR bluetooth OR "smart device" OR "smart devices" OR "automated reminder" OR "automated reminders" OR "synchronous video" OR "live video" OR "live videos" OR "remote patient monitoring" OR "home monitor" OR "home monitors" OR "home monitoring" OR telerounding OR teleround OR telerounds OR "tele rounds" OR "tele rounding" OR telehomecare OR telehome OR "tele home care" OR "tele homecare" OR "tele home health" OR televisit OR televisits OR "tele visit" OR "tele visits" OR teleradiology OR "tele radiology" OR teledermatology OR "tele dermatology" OR teleneurology OR "tele neurology" OR telepharmacy OR "tele pharmacy" OR "telemental health" OR "tele mental health" OR "tele behavioral" OR teleophthalmology OR "tele ophthalmology" OR telepathology OR "tele pathology" OR telediagnosis OR "tele diagnosis" OR telediagnoses OR telementor OR telementoring OR telementors OR "tele mentor" OR "tele mentoring" OR "virtual healthcare" OR "virtual health care" OR "digital health care" OR "digital healthcare" OR telepresence OR evisit OR evisits OR "e visit" OR "e visits" OR eicu OR "e ICU" OR teledentistry OR "remote screening" OR "internet-based intervention*" OR "web-based intervention*" OR "online intervention*" OR "tele-triage" OR telemonitoring OR "tele-monitoring" OR telediagnos* OR "tele-diagnos*" OR telecare OR "tele-care" OR "virtual care" OR teleprevent* OR "e-prescription" OR gamification OR ghealth OR "serious gam*" OR "Digital technolog*" OR "Digital electronic*" OR "mobile technolog*" OR "Portable Electronic App*" OR "Portable Software App*" OR "text-messag*" OR texting OR "Short message service" OR "Cell phone*" OR smartphone* OR "smart phone*" OR "i-Phone*" OR "i-Pad" OR (("intra-oral camera*" OR "oral camera*" OR "real-time*" OR "store-and-forward") W/4 (remote OR distan*)))	1,511,862
2	TITLE-ABS-KEY (teledent* OR "tele-dent*")	506
1	TITLE-ABS-KEY ("Stomatognathic Disease*" OR "Mouth and Tooth Disease*" OR "Dental Disease*" OR dentist* OR dental OR caries OR periodont* OR "dental care" OR "mouth care" OR "dental hygiene" OR "dental health services" OR "oral health*" OR "oral hygiene*" OR "oral medicine*" OR "mouth disease*" OR "oral healthcare" OR "oral health care" OR "oral cancer*" OR "oral neoplasm*" OR "mouth cancer*" OR "oral leukoplakia" OR "lip cancer*" OR "lip neoplasm*" OR "tongue cancer*" OR "tongue neoplasm*" OR "salivary gland cancer*" OR "salivary gland neoplasm*" OR "oral lesion*" OR "oral ulcer*" OR "mouth ulcer*" OR "buccal ulcer*" OR "bacterial oral	952,906

	infection*" OR noma OR "fungal oral infection*" OR stomatitis OR "oral mucositis" OR "tooth decay" OR "tooth fissure*" OR "dental fissure*" OR "tooth disease*" OR "tooth loss*" OR "teeth loss*" OR edentulism OR edentulous OR toothless OR "lacking teeth" OR xerostomia OR hyposalivation OR "mouth dryness" OR "oral dryness" OR "oral health education" OR "oral health information" OR "oral health literacy" OR "oral health hygiene" OR toothbrush* OR "tooth brush*" OR flossing OR "oral pain" OR "dental pain*" OR "mouth pain" OR gingivitis OR "tooth wear" OR "tooth erosion*" OR "tooth abrasion*" OR "dental abrasion*" OR "dental erosion*" OR "tooth abscess*" OR "oral abscess*" OR "dental abscess*" OR "oral cyst*" OR "access to oral care" OR "access to dental care")	

Key: TITLE-ABS-KEY = searches in title, abstract and author supplied keywords, EMTREE subject headings, other keywords, trade names and chemical names.

	Cochrane Database of Systematic Reviews (Wiley) from inception to April 24th 2023	
ID	Search	Hits
#1	("Stomatognathic Disease" OR “Stomatognathic diseases” OR "Mouth and Tooth Disease" OR “Mouth and Tooth Diseases” OR "Dental Disease" OR “Dental Diseases” OR "dentist*" OR dental OR caries OR periodont* OR "dental care" OR "mouth care" OR "dental hygiene" OR "dental health services" OR "oral health" OR "oral hygiene" OR "oral medicine" OR "mouth disease" OR “mouth diseases” OR "oral healthcare" OR "oral health care" OR "oral cancer" OR “oral cancers” OR "oral neoplasm" OR “oral neoplasms” OR "mouth cancer" OR “mouth cancers” OR "oral leukoplakia" OR "lip cancer" OR “lip cancers” OR "lip neoplasm" OR “lip neoplasms” OR "tongue cancer" OR “tongue cancers” OR "tongue neoplasm" OR “tongue neoplasms” OR "salivary gland cancer" OR “salivary gland cancers” OR "salivary gland neoplasm" OR “salivary gland neoplasms” OR "oral lesion" OR “oral lesions” OR "oral ulcer" OR “oral ulcers” OR "mouth ulcer" OR “mouth ulcers” OR "buccal ulcer" OR “buccal ulcers” OR "bacterial oral infection" OR “bacterial oral infections” OR Noma OR "fungal oral infection" OR “fungal oral infections” OR stomatitis OR "oral mucositis" OR "tooth decay" OR "tooth fissure" OR “tooth fissures” OR "dental fissure" OR “dental fissures” OR "tooth disease" OR “tooth diseases” OR "tooth loss" OR “tooth losses” OR "teeth loss" OR “teeth losses” OR edentulism OR edentulous OR toothless OR "lacking teeth" OR xerostomia OR hyposalivation OR "mouth dryness" OR "oral dryness" OR "oral health education" OR "oral health information" OR "oral health literacy" OR "oral health hygiene" OR toothbrush* OR "tooth brush" OR “tooth brushes” OR “tooth brushing” OR flossing OR "oral pain" OR "dental pain*" OR "mouth pain" OR gingivitis OR "tooth wear" OR "tooth erosion" OR "tooth abrasion" OR “tooth abrasions” OR "dental abrasion" OR “dental abrasions” OR "dental erosion" OR "tooth abscess" OR “tooth abscesses” OR "oral abscess" OR “oral absesses” OR "dental abscess" OR “dental abscesses” OR "oral cyst" OR “oral cysts” OR "access to oral care" OR "access to dental care"):ti,ab,kw	60,154
#2	(teledent* OR “tele-dentist” OR “tele-dentistry” OR “tele-dental”):ti,ab,kw	18
#3	("mobile health" OR Telehealth* OR "m-health" OR "e-health" OR ehealth OR mhealth* OR telemedicine OR "tele-medicine" OR telepsychiatr* OR "tele-psychiatry" OR telepsychotherap* OR "tele-psychotherapy" OR "tele-health" OR "Remote consult" OR “remote consults” OR “remote consultations” OR teleconsult* OR "distance counseling" OR "e-therapy" OR “e-therapies” OR "e-counseling" OR Telecommunication* OR teleconferenc* OR "tele-conference" OR “tele-conferences” OR “teleconferencing” OR "digital health" OR telerehabilitation OR "tele-rehabilitation" OR "virtual rehabilitation" OR "e rehabilitation" OR erehabilitation OR "remote rehabilitation" OR teleexercise* OR "tele exercise" OR “tele-exercises” OR telenutrition OR "tele nutrition" OR "e nutrition" OR enutrition OR telenutrition OR "tele nutrition" OR etherap* OR "mobile tool*" OR "touch screen" OR “touch screens” OR "remote health care" OR "remote healthcare" OR "remote consulting" OR "remote delivery" OR "mobile technology" OR "computer intervention" OR “computer interventions” OR "internet based" OR "remote monitor" OR “remote monitoring” OR "tele consult" OR “tele consults” OR “tele consulting” OR teleconsult* OR "tele practice" OR “tele practices” OR landline* OR skype OR facetime OR "tablet device" OR “tablet devices” OR "mobile app" OR "mobile apps" OR "mobile application" OR “mobile applications” OR "palm pilot" OR pager OR pagers OR "mobile device" OR "mobile devices" OR "mobile phone" OR "mobile phones" OR "cellular phone" OR "cellular phones" OR "cellular device" OR "cellular devices" OR "tablet computer" OR "tablet computers" OR blackberry OR android OR laptop OR laptops OR "tablet personal computer" OR "tablet personal computers" OR Wii OR "store and forward" OR videoconference OR videoconferences OR videoconferencing OR "video conference" OR "video conferencing" OR "video conferences" OR "video consultation" OR "video consultations" OR wireless OR telemetry OR telemetric OR telemetrical OR biotelemetry OR biotelemetric OR biotelemetrical OR bluetooth OR "smart device" OR "smart devices" OR "automated reminder" OR "automated reminders" OR "synchronous video" OR "live video" OR	41,365

	"live videos" OR "remote patient monitoring" OR "home monitor" OR "home monitors" OR "home monitoring" OR telerounding OR teleround OR telerounds OR "tele rounds" OR "tele rounding" OR telehomecare OR telehome OR "tele home care" OR "tele homecare" OR "tele home health" OR televisit OR televisits OR "tele visit" OR "tele visits" OR teleradiology OR "tele radiology" OR teledermatology OR "tele dermatology" OR teleneurology OR "tele neurology" OR telepharmacy OR "tele pharmacy" OR "telemental health" OR "tele mental health" OR "tele behavioral" OR teleophthalmology OR "tele ophthalmology" OR telepathology OR "tele pathology" OR telediagnosis OR "tele diagnosis" OR telediagnoses OR telementor OR telementoring OR telementors OR "tele mentor" OR "tele mentoring" OR "virtual healthcare" OR "virtual health care" OR "digital health care" OR "digital healthcare" OR telepresence OR evisit OR evisits OR "e visit" OR "e visits" OR eICU OR "e ICU" OR teledentistry OR "remote screening" OR "internet-based intervention*" OR "web-based intervention*" OR "online intervention*" OR "tele-triage" OR "telemonitoring" OR "tele-monitoring" OR "telediagnos*" OR "tele-diagnos*" OR "telecare" OR "tele-care" OR "virtual care" OR "teleprevent*" OR "e-prescription" OR Gamification OR ghealth OR "serious game" OR “serious games” OR “serious gaming” OR "Digital technology" OR "Digital electronic" OR “digital electronics” OR "mobile technology" OR "Portable Electronic App" OR “portable electronic apps” OR "Portable Software App" OR “portable software apps” OR "text-message" OR “text-messages” OR “text-messaging” OR texting OR "Short message service" OR "cell phone" OR “cell phones” OR smartphone* OR "smart phone" OR “smart phones” OR "i-Phone" OR “i-Phones” OR "i-Pad"):ti,ab,kw	
#4	(("intra-oral camera" OR “inta-oral cameras” OR "oral camera" OR “oral camera” OR "real-time" OR "store-and-forward") AND (remote OR distan*)):ti,ab,kw	709
#5	#3 OR #4	41,676
#6	(#1 AND #5) OR #2	633
#7	#6 AND Cochrane Database of Systematic Reviews	63

Key: ti,ab,kw searches in title, abstract and author supplied keywords

	NHS EED + HTA (University of York, Centre for Reviews and Dissemination	
Line	Query	Hits
1	((Dentistry[Mesh] OR Oral Health[Mesh] OR Stomatognathic Diseases[Mesh] OR Dental Clinics[Mesh] OR Stomatognathic OR Mouth OR Tooth OR Dental OR dentist OR dentistry OR caries OR periodontal OR oral health OR oral hygiene OR oral medicine OR oral healthcare OR oral cancer OR oral cancers OR oral neoplasm OR oral neoplasms OR lip cancer OR lip cancers OR lip neoplasm OR lip neoplasms OR tongue cancer OR tongue cancers OR tongue neoplasm OR tongue neoplasms OR salivary gland cancer OR salivary gland cancers OR salivary gland neoplasm OR salivary gland neoplasms OR oral lesion OR oral lesions OR oral ulcer OR oral ulcers OR buccal ulcer OR buccal ulcers OR bacterial oral infection OR bacterial oral infections OR Noma OR fungal oral infection OR fungal oral infections OR stomatitis OR oral mucositis OR teeth OR edentulism OR edentulous OR toothless OR xerostomia OR hyposalivation OR oral dryness OR toothbrush OR tooth brush OR tooth brushes OR tooth brushing OR flossing OR oral pain OR gingivitis OR oral abscess OR oral absesses OR oral cyst OR oral cysts OR oral care)) and ((Economic evaluation:ZDT and Bibliographic:ZPS) OR (Economic evaluation:ZDT and Abstract:ZPS) OR Project record:ZDT OR Full publication record:ZDT) IN NHSEED, HTA	437
2	((teledentistry OR tele-dentistry OR teledental OR tele-dental OR teledentist OR tele-dentist)) and ((Economic evaluation:ZDT and Bibliographic:ZPS) OR (Economic evaluation:ZDT and Abstract:ZPS) OR Project record:ZDT OR Full publication record:ZDT) IN NHSEED, HTA	0
3	((Telemedicine[Mesh] OR Remote consultation[Mesh] OR Distance Counseling[Mesh] OR Telecommunications[Mesh] OR Internet-based intervention[Mesh] OR Telenursing[Mesh] OR Digital technology[Mesh] OR Mobile applications[Mesh] OR Text-messaging[Mesh] OR Cell phone[Mesh] OR Smartphone[Mesh] OR Electronic Health Records[Mesh] OR Videoconferencing[MeSH] OR Teleradiology[MeSH] OR Telepathology[MeSH] OR Mobile OR telemedicine OR teleconsult OR telehealth OR mhealth OR ehealth OR remote OR distance OR digital OR internet OR landline OR skype OR facetime OR device OR app OR phone OR smartphone OR blackberry OR android R computer OR video OR laptop OR wii OR virtual OR eICU OR webbased OR ghealth OR gamification OR online OR games OR texting)) and ((Economic evaluation:ZDT and Bibliographic:ZPS) OR (Economic evaluation:ZDT and Abstract:ZPS) OR Project record:ZDT OR Full publication record:ZDT) IN NHSEED, HTA	2767
4	#1 AND #3	24

	JBI (Database of systematic reviews and implementation report + Evidence synthesis) From 2012 to April 24th 2023	
Set	Query	Results
#4	#1 AND #3	1
#3	(Telemedicine OR Telecommunications OR Telenursing OR Text-messaging OR Electronic Health Records OR Videoconferencing OR Teleradiology OR Telepathology OR Mobile OR teleconsult OR telehealth OR mhealth OR ehealth OR remote OR distance OR digital OR internet OR landline OR skype OR facetime OR device OR app OR phone OR smartphone OR blackberry OR android R computer OR video OR laptop OR wii OR virtual OR eICU OR webbased OR ghealth OR gamification OR online OR games OR texting)	3,280
#2	teledentistry OR teledental OR teledentist	0
#1	(Stomatognathic OR Dental OR Mouth OR dentist OR dentistry OR caries OR periodontal OR oral OR lip OR tongue OR salivary OR buccal OR Noma OR stomatitis OR teeth OR edentulism OR edentulous OR toothless OR xerostomia OR hyposalivation OR toothbrush OR tooth OR flossing)	667
